# Genome sequence of *Equine Erythroparvovirus 1*, identified in the United States

**DOI:** 10.1128/mra.00897-24

**Published:** 2025-01-29

**Authors:** Y. Tina Yu, Ximena Olarte Castillo, Guillaume Reboul, Jordan Zehr, Yining Sun, Renee Anderson, Minghui Wang, Qi Sun, Rebecca Tallmadge, Kelly Sams, Joel Brown, Nicholas Marra, Bryce Stanhope, Jennifer Grenier, Colin R. Parrish, Nicola Pusterla, Thomas Divers, Linda Mittel, Laura B. Goodman

**Affiliations:** 1 College of Veterinary Medicine, Cornell University, Ithaca, New York, USA; 2 Center for Biotechnology, Cornell University, Ithaca, New York, USA; 3 Division of Science, Mathematics, and Technology, Governors State University, University Park, Illinois, USA; 4 University of California, Davis, California, USA; Katholieke Universiteit Leuven, Leuven, Belgium

**Keywords:** genomes, parvovirus, taxonomy, veterinary clinical studies

## Abstract

*Equine Erythroparvovirus 1* is a parvovirus that was identified in the blood of four horses in the United States. Here, we report one genome from a horse in New York State. This genome may represent a new species within the genus *Erythroparvovirus*.

## ANNOUNCEMENT

Parvoviruses contain a linear single-stranded DNA genome of ∼5 kb (1). *Erythroparvovirus*, a genus within the family *Parvoviridae*, has been reported primarily in the sera of primates, seals, bovines, and rodents, but not in equines (2). The viral genome contains two open reading frames: NS and VP. The NS1 gene is largely responsible for DNA replication, DNA damage response, and apoptosis pathways. According to Cotmore et al.’s proposal (3), viruses within the family *Parvoviridae* are considered the same species if the NS1 proteins share >85% amino acid sequence identity while diverging by >15% from other virus species of the same genus. Many parvoviruses are considered to be nonpathogenic (4).

Through metagenomic sequencing, an *Erythroparvovirus* was detected in a blood sample from an observational study on equine fevers in November 2012 (Y. Sun, submitted for publication). The horse (150458) was a 13-year-old gelding with a fever of 101.5°F and no other clinical signs.

A blood sample was collected as part of routine clinical care, and 200 µL was treated with ZAP-OGLOBIN II Lytic Reagent at 37°C for 15 min (NC0098316, Beckman Coulter), then extracted using the MagMAX CORE Nucleic Acid Purification Kit (A32700, Thermo-Fisher). Library preparation was performed using the Illumina DNA Prep kit and sequenced using Illumina MiSeq paired-end 2*250 setting on v3 chemistry. The raw data contained 1,315,288 total reads. Sequences were then quality-checked and cleaned using trimmomatic v.0.39 (5), retaining trimmed sequences above 100 nucleotides long. Paired-end sequences retained after trimming and removing low-quality reads were used for assembly using SPAdes version 3.15.5 (6) with—meta (7) with default options. Other contigs containing non-parvovirus sequences were removed from the resulting file, and a fasta file with solely the putative parvovirus genome contig was created using awk. Prokka (8) v.1.14.5-r9 was used with option—kingdom set to “Viruses” to annotate the putative parvovirus genome. The genome showed 76.1% similarity to Primate Erythroparvovirus 1 (*Human parvovirus B19*) and 75.2% similarity to Ungulate Erythroparvovirus 1 (*Bovine parvovirus 3*), which, despite having a lower *e*-value (2e−32 compared to 8e−18 for Human B19), showed a slightly lower percentage identity of 75.2%.

The genome has a GC content of 56.6% and size of 5,115 bp. The putative NS1 gene is identified as spanning from nucleotides 2,736 to 4,967, and the putative VP gene from nucleotides 53 to 2,731. The missing number of bases at the 5′ and 3′ ends of the genome could be due to limitations of read length of short-read untargeted sequencing, making the ends of the genome less represented, leading to gaps in the region. The closest relative for the full NS1 gene is *Bovine parvovirus 3*/*Erythroparvovirus ungulate 1* (MG745680) with 75.3% nucleotide identity. The closest viral NS1 amino acid match was *Chipmunk parvovirus* (YP_009507375.1) with 49.25% identity, 61% query coverage based on a BLASTP search of the refseq_protein database. Expanding the search to the nr protein database resulted in a match to a metagenome-assembled genome of *Parvovirus panthera NS1* (DBA48951.1) with a better query coverage (83%) and 45.5% identity. Our findings suggest that the constructed *Erythroparvovirus* genome is very likely a new *Erythroparvovirus* species.

## Data Availability

The assembled genome is deposited in GenBank under accession PP681139. The raw reads are deposited in SRA under accession SRX23874400.

## References

[1] Cotmore SF, Tattersall P. 2014. Parvoviruses: small does not mean simple. Annu Rev Virol 1:517–537. doi:10.1146/annurev-virology-031413-085444 26958732

[2] Jager MC, Tomlinson JE, Lopez-Astacio RA, Parrish CR, Van de Walle GR. 2021. Small but mighty: old and new parvoviruses of veterinary significance. Virol J 18:210. doi:10.1186/s12985-021-01677-y 34689822 PMC8542416

[3] Cotmore SF, Agbandje-McKenna M, Chiorini JA, Mukha DV, Pintel DJ, Qiu J, Soderlund-Venermo M, Tattersall P, Tijssen P, Gatherer D, Davison AJ. 2014. The family parvoviridae. Arch Virol 159:1239–1247. doi:10.1007/s00705-013-1914-1 24212889 PMC4013247

[4] Qiu J, Söderlund-Venermo M, Young NS. 2017. Human parvoviruses. Clin Microbiol Rev 30:43–113. doi:10.1128/CMR.00040-16 27806994 PMC5217800

[5] Bolger AM, Lohse M, Usadel B. 2014. Trimmomatic: a flexible trimmer for Illumina sequence data. Bioinformatics 30:2114–2120. doi:10.1093/bioinformatics/btu170 24695404 PMC4103590

[6] Prjibelski A, Antipov D, Meleshko D, Lapidus A, Korobeynikov A. 2020. Using SPAdes de Novo assembler. CP in Bioinformatics 70:e102. doi:10.1002/cpbi.102 32559359

[7] Nurk S, Meleshko D, Korobeynikov A, Pevzner PA. 2017. metaSPAdes: a new versatile metagenomic assembler. Genome Res 27:824–834. doi:10.1101/gr.213959.116 28298430 PMC5411777

[8] Seemann T. 2014. Prokka: rapid prokaryotic genome annotation. Bioinformatics 30:2068–2069. doi:10.1093/bioinformatics/btu153 24642063

